# The correlation between the levels of tissue inhibitor of metalloproteinases 1 in plasma and tumour response and survival after preoperative radiochemotherapy in patients with rectal cancer

**DOI:** 10.2478/raon-2013-0028

**Published:** 2013-05-21

**Authors:** Irena Oblak, Vaneja Velenik, Franc Anderluh, Barbara Mozina, Janja Ocvirk

**Affiliations:** 1 Department of Radiotherapy, Institute of Oncology Ljubljana, Ljubljana, Slovenia; 2 Department of Laboratory Diagnostics, Institute of Oncology Ljubljana, Ljubljana, Slovenia; 3 Department of Medical Oncology, Institute of Oncology Ljubljana, Ljubljana, Slovenia

**Keywords:** tissue inhibitor of metalloproteinases, rectal cancer, preoperative radiochemotherapy, prognostic factors

## Abstract

**Background:**

The aim of this study was to analyse whether the level of tissue inhibitor of metalloproteinases (TIMP) 1 is associated with the tumour response and survival to preoperative radiochemotherapy in rectal cancer patients.

**Patients and methods.:**

Ninety-two patients with histologically confirmed non-metastatic rectal cancer of clinical stage I– III were treated with preoperative radiochemotherapy, surgery and postoperative chemotherapy. Plasma TIMP-1 concentrations were measured prior to the start of the treatment with an enzyme-linked immunosorbent assay (ELISA).

**Results:**

Median follow-up time was 68 months (range: 3–93 months) while in survivors it was 80 months (range: 68–93 months). The 5-year locoregional control (LRC), disease-free survival (DFS), disease-specific survival (DSS) and overall survival (OS) rates for all patients were 80.2%, 56.4%, 63.7% and 52.2%, respectively. The median TIMP-1 level was 185 ng/mL (range: 22–523 ng/mL) and the mean level (±standard deviation) was 192 (±87) ng/mL. Serum TIMP-1 levels were found to be significantly increased in patients with preoperative CRP>12 mg/L and in those who died from rectal cancer or had cT4 tumours. No correlation was established for age, gender, carcinoembriogenic antigene (CEA) level, platelets count, histopathological grade, response to preoperative therapy, resectability and disease reappearance. On univariate analysis, various parameters favourably influenced one or more survival endpoints: TIMP-1 <170 ng/mL, CRP <12 mg/L, platelets count <290 10E9/L, CEA <3.4mg/L, age <69 years, male gender, early stage disease (cN0 and/or cT2–3), radical surgery (R0) and response to preoperative radiochemotherapy. In multivariate model, LRC was favourably influenced by N-downstage, DFS by lower CRP and N-downstage, DSS by lower CRP and N-downstage and OS by lower TIMP-1 level, lower CRP and N-downstage.

**Conclusions:**

Although we did not find any association between pretreatment serum TIMP-1 levels and primary tumour response to preoperative radiochemotherapy in our cohort of patients with rectal cancer, TIMP-1 levels were recognized as an independent prognostic factor for OS in these patients.

## Introduction

Rectal cancer is one of the major health problems due to increasing incidence and in a substantial proportion of patients it is diagnosed in advanced stages. The primary treatment is surgery. In transmural extension of tumour or node positive disease, preoperative radiochemotherapy is recommended to improve local control and survival.^1^–^3^

Poor prognosis of rectal cancer patients was attributed to the increase in tumour aggressiveness and its metastatic potential which, among others, were found to be determined by the activity of matrix metalloproteinases (MMPs) and their inhibitors (TIMPs) which play an important role in the process of degradation of the extracellular matrix and the basal membranes. The activity of the MMPs depends on the balance between the level of the active enzyme and its inhibitor.^4^–^7^ The TIMP family consists of 4 members: TIMP-1, TIMP-2, TIMP-3 and TIMP-4, which act as negative regulators of the degradation processes in the extracellular matrix. The latest evidences suggest that they also participate in cell signalling by regulating cell growth, apoptosis, angiogenesis and genomic instability.^6^,^8^,^9^

By analysing TIMP (and TIMP-1 in particular) expression in tumour tissue or circulating TIMP levels in plasma, they were recognized as candidate markers for diagnosis and prognosis in patients with different types of cancer, *e.g*. colorectal, breast, lung, ovarian, bladder carcinoma^6^,^9^, but, also in a variety of nonmalignant conditions: asthma, diabetes, cardiovascular and autoimmune diseases.^9^ Increased TIMP-1 plasma levels were found in patients with colorectal cancer compared to healthy controls, and were associated with advanced disease stage and poor prognosis.^6^,^8^–^12^ In metastatic colorectal cancer, elevated level of the inhibitor in plasma was predictive for low probability of response to chemotherapy.^13^ Finally, Unsal *et al*. reported that matrix metalloproteinase-9 expression correlates with a poor tumour response to preoperative chemoradiotherapy in patients with locally advanced rectal cancer.^14^

The aim of this study was to determine the predictive and prognostic value of TIMP-1 plasma level for local tumour response to preoperative radiochemotherapy and survival in patients with rectal cancer.

## Patients and methods

### Patients and treatment

Ninety-two patients (63 male, 29 female), aged from 42 to 86 years (mean 73 years), with histologically confirmed non-metastatic rectal cancer of clinical stage I– III, were included in prospective study. They were treated with preoperative radiochemotherapy at the Institute of Oncology in Ljubljana between March 2005 and October 2006. In majority of patients (76; 82.6%) tumours were staged as cT3 and clinically positive regional nodes were found in more than half of the cases (48; 52.2%) (Table 1). Patients underwent a general clinical examination, including rectal examination by two independent examiners, complete blood tests, chest radiography and abdominopelvic computer tomography (CT), rectoscopy, endoscopic ultrasound (US) and magnetic resonance imaging (MRI) of the pelvis.

Preoperatively, patients were irradiated using CT-based 3-dimensional (3D) planning and four-field box technique at a 15 MV linear accelerator. Radiotherapy regimen was comprised of 45 Gy in 25 fractions to small pelvis with a boost of 5.4 Gy to the primary tumour. In case of cT4 tumour or unresectable disease the booster dose was 9 Gy. Concurrently with irradiation all patients had chemotherapy (ChT), consisted of continuous peroral capecitabine (825 mg/m^2^ b.i.d., days 1–33, without weekend breaks) or of 2 cycles of intravenous 5-fluorouracil (5-FU) and leucovorine (425 mg/m^2^/day and 20 mg/m^2^/day, days 1 to 5).

Surgery employing total mesorectal excision (TME) technique was scheduled 6–8 weeks after the completion of chemoradiation and sphincter preservation procedure was done whenever possible. Four cycles of chemotherapy with either capecitabine (1250 mg/m^2^ bid, days 1–14, every 3 weeks) or bolus 5-FU and leucovorine (425 mg/m^2^/ day and 20 mg/m^2^/day, days 1–5, every 4 weeks) were planned postoperatively. The choice of postoperative chemotherapy was left to the oncologist’s discretion. After finishing all therapies, patients were followed in 3 month intervals for the first 2 years and thereafter every six months up to the 5th year.

### Determination of plasma TIMP-1 level

Blood samples were collected in vacutainer EDTA tubes. After centrifugation at 1500 × g at 4°C for 10 minutes, plasma was separated and kept at −80°C until analysis. Plasma TIMP-1 concentrations were measured with an enzyme-linked immunosorbent assay (ELISA) using commercially available TIMP-1 ELISA kit, purchased from Oncogen Science, Cambridge (MA), USA. Procedures were performed according to the manufacturer′s protocol in duplicate, using diluted samples. All measurements differed by less than 10% and the mean value was calculated and used for statistical analysis. The inter-assay precision coefficients of variation of TIMP-1 ranged between 3.9% and 8.8% at the different levels.

### Statistical analysis

Statistical analysis was performed using personal computer and software statistical package SPSS, version 13 (SPSS Inc., USA). The main endpoints of the study were response to preoperative therapy, locoregional control (LRC, the event was local or regional recurrence), disease-free survival (DFS, the event was local, regional or systemic recurrence), disease-specific survival (DSS, the event was death due to the rectal cancer) and overall survival (OS, the event was death from any cause). Mann-Whitney test for independent groups was used to assess the differences in plasma TIMP-1 levels and clinicopathological parameters between various groups of patients. The survival of patients was computed from the first day of therapy to the closure date (December 31^st^, 2012). The Kaplan-Meier estimate was employed to calculate survival probability^15^, and the differences between plotted curves were tested using the log-rank test.^16^ Independent prognostic value of factors that appeared statistically significant on univariate analysis was tested in multivariate Cox proportional hazard regression analysis.^17^ A two-tailed P < 0.05 was considered as statistically significant.

The study protocol was approved by the Medical Ethics Committee at the Ministry of Health of the Republic of Slovenia. All of the included patients gave their informed consent to the voluntary participation in the study.

## Results

### Course of treatment and outcome

Therapy as planned was delivered to 67 (70.8%) of patients: 16 (17.4%) patients had no concurrent ChT (history of ischaemic heart disease or other serious comorbidity) and in 9 (11.8%) patients it was omitted before the end of the preoperative treatment due to the acute side effects. Abdominoperineal excision, low anterior resection, and palliative surgery only was performed in 32 (34.8%), 52 (56.6%) and 4 (4.3%) patients, respectively. Four (4.3%) patients had no surgery (refusal 1, disease progression 3). Histopathologically radical resection (R0) was performed in 80 (87%) patients and pathological complete and partial response rates after preoperative radiochemotherapy were 16.3% and 34.8%, respectively. Postoperatively, adjuvant ChT was administered to 27 (29.3%) patients and 70.4% of them received 4 cycles.

Median follow-up time was 68 months (range: 3–93 months) and was 80 months (range: 68–93 months) in survivors. On the close-out date, 42 (45.7%) patients were alive, 33 (35.9%) patients died of rectal cancer whereas in 11 (12%) patients the causes of death were vascular events (8) and pneumonia, metachronous bronchus cancer and malignant melanoma in 1 patient each, but, in 6 patients the cause of death was unknown. Sites of failure were as follows: local/locoregional 9 (9.8%), distant 23 (25%), local/locoregional and distant 5 (5.5%). The 5-year LRC, DFS, DSS and OS rates for all patients were 80.2%, 56.4%, 63.7% and 52.2%, respectively.

### TIMP-1 and prognosis

The median TIMP-1 level was 185 ng/mL (range: 22–523 ng/mL) and the mean level (± standard deviation) was 192 (±87) ng/mL. Serum TIMP-1 levels were found to be significantly increased in patients with preoperative CRP>12 mg/L and in those who died from rectal cancer and were marginally increased in patients who had cT4 tumours. No correlations were established for age, gender, cN-stage, CEA level, platelets count, histopathological grade, response to preoperative therapy, resectability and disease re-appearance.

**FIGURE 1:**
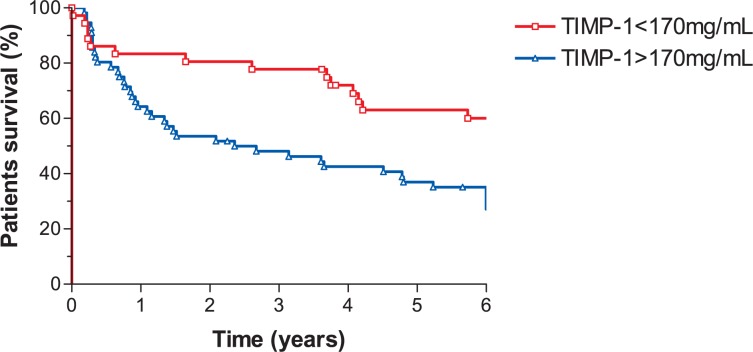
Overall survival and tissue inhibitor of metalloproteinases 1 (TIMP-1).

On univariate analysis, various parameters favourably influenced one or more survival endpoints: TIMP-1 < 170 ng/mL (Figure 1), CRP < 12 mg/L, platelets count < 290 10^9^ L, CEA < 3.4 mg/L, age < 69 years, male gender, early stage disease (cN0 and/or cT2-3), radical surgery (R0) and response to preoperative radiochemotherapy (Table 2). The following parameters were introduced in multivariate model to test their independent prognostic value: CRP, T-downstage, N-downstage and pretreatment TIMP-1 level. LRC was favourably influenced by N-downstage (Hazard ratio [HR] = 6.10; 95% confidence interval [CI]: 1.78–20.89; *p* = 0.004); DFS by CRP (HR = 3.09; 95% CI: 1.33–7.18; *p* = 0.009) and N-downstage (HR = 3.66; 95% CI: 1.58–8.52; *p* = 0.003); DSS by CRP (HR = 2.77; 95% CI: 1.13–6.76; *p* = 0.03) and N-downstage (HR = 3.88; 95% CI: 1.62–9.32; *p*=0.002); OS by TIMP-1 level (HR = 2.15; 95% CI: 1.01–4.56; *p* = 0.047), CRP (HR = 2.14; 95% CI: 1.08–4.25; *p*=0.029) and N-downstage (HR = 2.89; 95% CI: 1.35–6.18; *p* = 0.006) (Table 3).

## Discussion

Several authors reported on the positive correlation between elevated serum or tissue TIMP-1 levels and increased aggressiveness of the disease. We can assume that elevated TIMP-1 levels reflect the degree of proteolytic activity which is an essential process implicated in invasiveness of tumour cells. Therefore, it was hypothesized that if TIMP-1 is predictive for relapse and survival^6^,^8^,^10^,^11^,^18^,^19^ it could be used to distinguish between patients with higher and those with lower risk for the disease recurrence. Detailed knowledge on risk level for disease re-appearance would allow us to avoid over- or undertreatment. Furthermore, the ability to predict efficiency of specific type of therapy, *e.g*. preoperative radiochemotherapy in rectal cancer patients, could help us to tailor the entire “treatment package” more according to individual tumour characteristics which are usually not taken into consideration.

In this view, our intention was to assess the predictive value of serum TIMP-1 levels in cohort of patients with rectal cancer who were treated with preoperative radiochemotherapy. The studied population was representative in regards to the treatment results such as percentage of radical resections (87%), T- and N-downstaging (34.8%), pathological complete responses (16.3%) and survivals (at 5 years: LRC-80.2%, DFS-56.4%, DSS-63.7%, and OS-52.2%) which are from all aspects comparable with the results of other researchers.^20^,^21^

When analysing the association between established clinicopathological parameters and TIMP-1, we found elevated TIMP-1 levels in patients with higher cT-stage and those who died from rectal cancer or had increased CRP before the start of the preoperative treatment. In several studies, higher TIMP-1 levels were associated with advanced stage of the disease and poor prognosis.^6^,^10^,^11^,^22^ On the other hand, Holten-Anderson *et al*. did not find any differences in the TIMP-1 levels between Dukes stage A, B or C; patients with Dukes D rectal cancer, however, had significantly increased TIMP-1 level compared to less advanced stages.^23^ In many studies TIMP-1 was reported to be in positive correlation with patients’ age^6^,^10^,^22^,^24^, although Tayebjee *et al*. and our study found just the opposite.^25^ The relationship between TIMP-1 and CRP can be explained with the fact that they both participate in the processes of inflammation. Frederiksen *et al*. who systematically followed TIMP-1 levels before and after the surgery suggested that prolonged recovery due to postoperative infections may contribute to prolonged increase of plasma TIMP-1 level.^9^

In the present study, no correlation between TIMP-1 levels and the response to preoperative radiochemotherapy was observed, although Sørensen *et al*. reported that TIMP-1 can be predictive for the response to chemotherapy in colon carcinoma^13^ and Unsal *et al*. found that positive MMP-9 expression correlated with poor tumour response in patients with locally advanced rectal cancer undergoing preoperative radiochemotherapy.^14^

We also analysed the relationship between TIMP-1 levels and CEA, which is well recognized and still the only recommended marker used in the diagnosis and follow-up of patients with colorectal cancer. As confirmed in several studies, increased pretreatment CEA levels reflect an advanced disease stage and are forecasting an increased risk of recurrence and poor prognosis.^26^ However, similar as Holten-Andersen *et al*.^23^ we also did not find any association between TIMP-1 and CEA levels.

In Cox multivariate analysis we confirmed that patients in whom N-downstaging resulted from preoperative radiochemotherapy had more favourable LRC, DFS, DSS and OS, but patients with T-downstage did not have better survival at all. Furthermore, in patients younger than 69 years we did not find better survival rates, which is opposite to the results of other studies.^27^,^28^ Finally, we found increased TIMP-1 levels (≥170mg/mL) to be an independent risk factor for worse OS (p = 0.047). This observations are consistent with the reports of other authors who also identified higher TIMP-1 levels as being a negative predictor of shorter survival.^6^,^8^–^13^,^18^,^19^,^23^ To the opposite, Ishida *et al*. were not able to confirm any prognostic value of TIMP-1 for colorectal cancer patients.^24^

## Conclusions

Although we did not find any association between pretreatment serum TIMP-1 levels and primary tumour response to preoperative radiochemotherapy in our cohort of patients with rectal cancer, TIMP-1 levels were recognized as an independent prognostic factor for OS in these patients.

## Figures and Tables

**TABLE 1:** Characteristics of patients and their tumours (N=92)

Patients	
Sex (female/male)	29/63
Age	73 (42–86)^*^

Primary tumour site	
Lower rectum	30 (32.6%)
Middle rectum	44 (47.8%)
Upper rectum	18 (19.6%)

UICC cTNM-classification				
	N_0_	N_1_	N_2_	Total
T_1_				0
T_2_	4	3	0	7
T_3_	40	33	3	76
T_4_	0	6	3	9
Total	44	42	6	92

Overall stage	
Stage I	4 (4.3%)
Stage II	40 (43.5%)
Stage IIIA	3 (3.3%)
Stage IIIB	42 (45.6%)
Stage IIIC	3 (3.3%)

Histopathological grading	
Well differentiated, G_1_	14 (15.2%)
Moderately differentiated, G_2_	59 (64.1%)
Poorly differentiated G_3_	19 (20.7%)

* Median (range), in years

UICC = International Union Against Cancer; cTNM = clinical stage tumour-nodes-metastases

**TABLE 2:** Tissue inhibitor of metalloproteinases (TIMP-1) and clinicopathological variables

**Clinicopathological variables**	**N=92**	**TIMP-1 level in ng/mL Median (range)**	***p*-value**
Age			
< 69 years	37	172 (55–350)	NS
≥ 69 years	55	204 (22–523)	
Gender			
Male	63	183 (22–421)	NS
Female	29	200 (79–523)	
cT			
cT2+3	83	187 (22–421)	0.084
cT4	9	227 (129–523)	
cN			
cN0	44	172 (66–318)	NS
cN+	48	190 (22–523)	
Resectability			
R0	80	185 (22–523)	NS
R1+R2	8	183 (82–263)	
pT			
pT0+1+2	32	187 (22–421)	NS
pT3+4	56	183 (66–523)	
pN			
pN0	66	187 (22–362)	NS
pN+	22	183 (82–523)	
T- downstage			
Yes	32	190 (22–523)	NS
No	56	175 (66–350)	
N- downstage			
Yes	72	190 (22–523)	NS
No	16	168 (82–255)	
pCR			
Yes	15	182 (80–421)	NS
No	73	187 (22–523)	
CEA (mg/L)			
< 3.4	44	187 (22–523)	NS
< 3.4	48	185 (66–350)	
Platelets count (10E9/L)			
< 290	40	185 (55–421)	NS
< 290	52	190 (22–523)	
CRP (mg/L)			
< 12	73	175 (22–421)	0.031
≥ 12	19	227 (92–523)	
Recurrence			
Yes	37	187 (66–523)	NS
No	55	190 (22–421)	
Death from rectal cancer			
Yes	33	200 (66–523)	NS
No	59	172 (22–421)	
Dead			
Yes	50	207 (55–523)	0.02
No	42	167 (22–362)	

NS = not significant; pCR = pathologic complete remission; CEA = carcinoembriogenic antigene, CRP = C reactive protein

**TABLE 3:** Multivariate analysis of survival

**Parameter**	**Locoregional control**			**Disease-free survival**			**Disease-specific survival**			**Overall survival**		

	**HR**	**95% CI**	**P-VALUE**	**HR**	**95% CI**	**P-vALUE**	**HR**	**95% CI**	**P-VALUE**	**HR**	**95% CI**	**P-VALUE**
**TIMP-1** < 170 ng/mL/ ≥ 170 ng/mL	1.33	0.33–5.31	NS	1.16	0.50–2.72	NS	1.34	0.53–3.37	NS	2.15	1.01–4.56	0.047
**T-downstage** Yes/No	2.76	0.59–12.99	NS	1.93	0.82–4.57	NS	1.72	0.71–4.17	NS	1.55	0.79–3.01	NS
**N-downstage** Yes/No	6.10	1.78–20.89	0.004	3.66	1.58–8.52	0.003	3.88	1.62–9.32	0.002	2.89	1.35–6.18	0.006
**CRP** < 12 mg/L / ≥ 12 mg/L	2.75	0.68–11.17	NS	3.09	1.33–7.18	0.009	2.77	1.13–6.76	0.03	2.14	1.08–4.25	0.029

HR = hazard ratio; CI-confidence interval; NS = not significant
